# Efficacy of Anamorelin, a Novel Non-Peptide Ghrelin Analogue, in Patients with Advanced Non-Small Cell Lung Cancer (NSCLC) and Cachexia—Review and Expert Opinion

**DOI:** 10.3390/ijms19113471

**Published:** 2018-11-05

**Authors:** David C. Currow, Matthew Maddocks, David Cella, Maurizio Muscaritoli

**Affiliations:** 1 IMPACCT—Improving Palliative, Aged and Chronic Care through Clinical and Translational Research, Faculty of Health, University of Technology Sydney, Ultimo, NSW 2007, Australia; David.Currow@uts.edu.au; 2 Cicely Saunders Institute of Palliative Care, Policy & Rehabilitation, King’s College London, London SE5 9RJ, UK; matthew.maddocks@kcl.ac.uk; 3 Department of Medical Social Sciences and Robert H. Lurie Comprehensive Cancer Center, Northwestern University Feinberg School of Medicine, Chicago, IL 60611, USA; d-cella@northwestern.edu; 4 Department of Translational and Precision Medicine (formerly Department of Clinical Medicine), Sapienza University of Rome, 00185 Rome, Italy

**Keywords:** ghrelin, anamorelin, ROMANA 1, ROMANA 2, anorexia, non-small cell lung cancer

## Abstract

Cancer cachexia is a multilayered syndrome consisting of the interaction between tumor cells and the host, at times modulated by the pharmacologic treatments used for tumor control. Key cellular and soluble mediators, activated because of this interaction, induce metabolic and nutritional alterations. This results in mass and functional changes systemically, and can lead to increased morbidity and reduced length and quality of life. For most solid malignancies, a cure remains an unrealistic goal, and targeting the key mediators is ineffective because of their heterogeneity/redundancy. The most beneficial approach is to target underlying systemic mechanisms, an approach where the novel non-peptide ghrelin analogue anamorelin has the advantage of stimulating appetite and possibly food intake, as well as promoting anabolism and significant muscle mass gain. In the ROMANA studies, compared with placebo, anamorelin significantly increased lean body mass in non-small cell lung cancer (NSCLC) patients. Body composition analysis suggested that anamorelin is an active anabolic agent in patients with NSCLC, without the side effects of other anabolic drugs. Anamorelin also induced a significant and meaningful improvement of anorexia/cachexia symptoms. The ROMANA trials have provided unprecedented knowledge, highlighting the therapeutic effects of anamorelin as an initial, but significant, step toward directly managing cancer cachexia.

## 1. Cancer Cachexia, Ghrelin, and Anamorelin

Cancer cachexia is a multifactorial condition, usually defined as ≥5% weight loss during the six months prior to screening, or body mass index (BMI) <20 kg/m^2^ [1] in the presence of uncontrolled cancer, and is not reversible by nutrition alone. The etiology of cancer cachexia is not yet fully clarified; central features comprise anorexia, reduced food intake, and increased systemic inflammation (C-reactive protein levels above upper limits of normal) [2,3]. Pathophysiologic manifestations of cancer cachexia include a negative protein and energy balance, sarcopenia [4], and abnormal metabolism and progressive functional impairment [1]. Thus, relative to patients without cachexia, patients with cancer cachexia frequently experience greater symptom burden, reduced tolerance and responsiveness to chemotherapy, decrease in quality of life (QOL), and shortened survival time [5,6,7,8]. Weight loss and BMI are independent prognostic factors in patients with cancer and associated cachexia. The more accentuated the weight loss and the more rapid the decrease in BMI, the stronger the correlation with higher morbidity and mortality [9].

This debilitating condition develops in patients with various types of cancer, with a prevalence of 50–80%, depending on tumor type [10]. Patients with advanced non-small cell lung cancer (NSCLC) and cachexia also present with respiratory complications that compound the symptom burden of cachexia, including debilitating fatigue [11]. In these patients, cancer cachexia reaches a prevalence of 60% over the course of the disease [12].

There is no standard of care for the management of cancer cachexia. The ideal drug for cancer cachexia would improve appetite, food intake, and sense of well-being, while also attenuating muscle protein breakdown [13] and stimulating anabolism to increase lean body mass (LBM) and body weight without unacceptable side effects. Currently used drugs, such as corticosteroids and progestational agents, have been shown to enhance appetite and increase body weight [14,15,16], but have little, if any, positive effect on LBM and skeletal muscle [17,18]. Increases in body weight with these agents are mainly led by increases in fat mass (FM) and water retention, with weight gain and appetite stimulation reportedly transient and disappearing within weeks. In addition, both corticosteroids and progestational agents are associated with substantial toxicity. The most notable problem is that corticosteroids are catabolic and, as such, can contribute to skeletal muscle wasting [13,19,20]. This is the opposite of the desired effect and suggests that appetite stimulation in this setting comes at a high health cost. For corticosteroids, the long-term adverse events (AEs) include insulin resistance, fluid retention, steroidal myopathy, skin fragility, adrenal insufficiency, immunosuppression, and psychotropic effects [21,22]. For progestational agents, prolonged administration increases the risk of thromboembolic events [23,24], adrenal insufficiency [25], fluid retention, hypogonadism, and death [23].

International guidelines committees, including the European Society for Clinical Nutrition and Metabolism, the European Palliative Care Research Collaborative, and the National Comprehensive Cancer Network, consider reduced food intake and metabolic imbalances as central to cancer cachexia, and their treatment recommendations focus primarily on maintaining body weight [22,26,27]. Guidelines suggest a multimodal approach to treating cancer cachexia that comprises nutritional intervention, physical exercise to support maintenance of function [28,29], and pharmacologic treatment, although very limited data concerning the efficacy of this proposed strategy are currently available [30].

### Appetite-Improving Drugs: Ghrelin and Ghrelin Agonists

Ghrelin, a peptide gastric hormone, is the endogenous ligand of the growth hormone (GH) secretagogue receptor 1a and stimulates multiple pathways that regulate appetite, LBM, body weight, and metabolism [31,32]. In patients with advanced cancer, ghrelin has been shown to stimulate GH, significantly enhance appetite, and regulate energy balance, with minimal reports of drug-related AEs [33,34,35]. However, the parenteral administration of ghrelin, combined with its short half-life (less than 30 min), has considerably limited its clinical utility.

Anamorelin is a novel, orally active, highly selective non-peptide ghrelin analogue that has been shown to simultaneously target multiple components of cancer cachexia, including appetite, body composition, adipose tissue metabolism, energy expenditure, and inflammation [36,37]. The international, randomized, double-blind, placebo-controlled phase 3 trials ROMANA 1 (NCT01387269) and ROMANA 2 (NCT01387282) assessed the efficacy and safety of anamorelin in patients with advanced NSCLC and cachexia over a 12-week period [38] (Table 1). Anamorelin treatment was well tolerated and, compared with placebo, significantly increased the co-primary endpoint of LBM in both ROMANA 1 (median increase of 0.99 kg [95% confidence interval (CI): 0.61, 1.36] compared with median loss of 0.47 kg [95% CI: −1.00, 0.21]; *p* < 0.0001) and ROMANA 2 (0.65 kg gain [95% CI: 0.38, 0.91] compared with median loss of 0.98 kg [95% CI: −1.49, −0.41]; *p* < 0.0001), although it had no significant effect on the other co-primary endpoint, handgrip strength. In both studies, anamorelin versus placebo significantly improved total body weight (ROMANA 1: 2.20 ± 0.33 kg vs. 0.14 ± 0.36 kg, *p* < 0.0001; ROMANA 2: 0.95 ± 0.39 kg vs. −0.57 ± 0.44 kg, *p* < 0.0001), as well as LBM, FM, appendicular LBM, and total body mass [38]. These results are in line with anamorelin’s properties as a non-peptide ghrelin analogue. Anorexia/cachexia symptoms and concerns of patients were also significantly improved following anamorelin treatment (mean change in the Functional Assessment of Anorexia/Cachexia Therapy [FAACT] Anorexia/Cachexia Subscale [A/CS] [39] domain score, ROMANA 1: 4.12 ± 0.75 vs. 1.92 ± 0.81, *p* = 0.0004; ROMANA 2: 3.48 ± 0.94 vs. 1.34 ± 1.03, *p* = 0.0016) [38,40].

Participants with an Eastern Cooperative Oncology Group performance status ≤2 who completed dosing in either of the two original 12-week trials could enroll in a 12-week safety extension study, ROMANA 3 (NCT01395914). Over the ROMANA 3 treatment period, anamorelin continued to be well tolerated and significantly increased body weight, when compared with placebo, over the entire 24-week period (least-squares mean change ± standard error: 3.1 ± 0.6 kg vs. 0.9 ± 0.7 kg; *p* < 0.0001) [41]. Improvements in anorexia were also observed over the 24-week period (significant differences with anamorelin vs. placebo at weeks 3, 6, 9, 12, and 16 [*p* < 0.05]) [41].

In the clinical setting, a noteworthy response to nutritional support is observed in patients with severe undernutrition (BMI <20 kg/m^2^ at baseline) [1,18,42]. A retrospective post hoc analysis of pooled efficacy data in different subgroups of patients from ROMANA 1 and ROMANA 2 found that, compared with placebo, anamorelin led to greater improvements in body weight in patients with BMI <20 kg/m^2^ at baseline (treatment difference with anamorelin vs. placebo: 3.09 kg [95% CI: 1.73, 4.44]; *p* < 0.001). Importantly, anamorelin’s effect on body weight was even more pronounced in these low-BMI patients than in the pooled overall population (treatment difference: 2.19 kg [95% CI: 1.56, 2.83]; *p* < 0.001) [43].

The proportion of patients achieving ≥5% increase in body weight following anamorelin treatment was also assessed in the pooled overall population and in patients with BMI <20 kg/m^2^ at baseline. This threshold was chosen on the basis of the consideration that an unintended weight loss of ≥5% represents one of the diagnostic criteria for cancer anorexia/cachexia [1]. Interestingly, 34.1% of patients in the overall efficacy population and 47.3% of patients with BMI <20 kg/m^2^ at baseline benefited from anamorelin [43], compared with 13.4% and 17.4%, respectively, in the placebo arm. These results highlight anamorelin’s capacity to mimic ghrelin’s body weight-enhancing properties [34,35].

Patients with severe weight loss are an at-risk population [44] that is extremely difficult to treat [45]. As such, these results are of crucial clinical importance, as they demonstrate that anamorelin is highly effective in severely underweight patients who are at the greatest risk from cancer cachexia.

## 2. Relevance of Improvement in LBM, FM, and Handgrip Strength

### 2.1. Relevance of Improvement in LBM

The loss of LBM and the accompanying decline in physical function are cardinal features of cancer cachexia. An assessment approach that considers body composition is important for patients with lung cancer and cachexia, as neither body weight [46,47] nor BMI [48] are strongly correlated with LBM or skeletal muscle.

A decline in LBM is observed as a natural feature of aging, with LBM reported to decrease from 50% of total body weight in healthy young adults to about 25% by the age of 75–80 years [49,50]. The natural decline typically commences in the sixth decade of life and is very gradual, with a mean loss of only 6–8% per decade, which equates to about 2.5–2.8 kg, depending on stature [51]. In patients with cancer, this decline is accelerated. Longitudinal studies report LBM loss between 0.14 and 0.20 kg per month [52] and, in patients with lung cancer, up to 6% over 100 days [53]. In the year preceding death, only 15% of patients achieve a measurable gain in muscle mass without intervention when assessed serially; muscle mass loss is more evident in patients with progressive compared with stable disease and is much more common in patients within three months of death [54].

**Figure 1:**
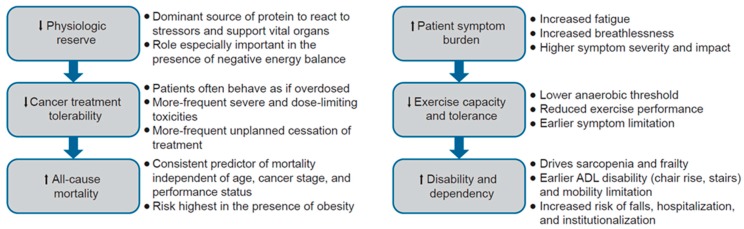
Clinical relevance of low LBM. ADL, activities of daily living; LBM, lean body mass.

LBM loss leads to reduced skeletal muscle function and performance (Figure 1). LBM has a role in whole-body metabolism, given that skeletal muscle forms the body’s dominant source of protein. Loss of LBM depletes this storage, diminishes physiologic reserves, and reduces the ability to withstand insult, particularly in response to stress [55,56]. This may explain why patients with low LBM tend to tolerate chemotherapy poorly, as the low muscle mass may lower the capacity for metabolizing and clearing drugs, leading to enhanced drug toxicity [57]. Across multiple cohort studies, patients with low LBM experience more severe or dose-limiting toxicities and earlier cessation of chemotherapy compared with those with relatively higher LBM [22]. Recent examples include cohorts of patients with newly diagnosed lung cancer (*N* = 134) [58], patients commencing first- or second-line chemotherapy (*N* = 200) [59], and patients scheduled for any anticancer treatment (*N* = 80) [57]. These findings are not limited to cancer type or anticancer drug, as low LBM is shown to predict toxicity in patients with various types of cancers treated with sorafenib [60], sunitinib [61], or afatinib [62]. Conversely, maintaining or gaining muscle mass during chemotherapy has been independently associated with reduced mortality, which may reflect stabilization of the disease, but also successful management of cancer cachexia [63]. Low LBM and low muscle mass have been associated with higher mortality in several studies with small- to medium-sized cohorts [22,58], although there is variation in the cutoff points used, most of which are generated retrospectively, and these studies generally lack a validation cohort [64].

Muscle dysfunction in cancer cachexia is driven by systemic inflammation and involves the peripheral and respiratory muscles. In patients with advanced lung cancer (*N* = 40), lower limb and inspiratory muscle strength are independently positively associated with whole-body exercise performance (r = 0.44 and 0.39, respectively) [65]. Conversely, loss of peripheral muscle results in buildup of waste products perceived as local muscle soreness or fatigue. These waste products also provide an afferent feedback to the respiratory centers to increase the rate of breathing [66]. With impaired respiratory muscle capacity, the body cannot respond appropriately to these afferent messages, and the resultant efferent–afferent mismatch is responsible for chronic breathlessness [67,68]. In a longitudinal study tracking muscle mass in advanced cancer (*N* = 368), gains in muscle mass were associated with improved physical function and ability to eat, better symptom control, and better response to anticancer treatment [54].

### 2.2. LBM and Handgrip Strength Responses to Interventions

The LBM response to various interventions in patients with advanced cancers is highly indicative of their efficacy and has been used as a primary outcome measure in a number of clinical trials, as described below.

In the two ROMANA trials, anamorelin treatment led to significant increases in LBM and an impressive arrest of ongoing weight loss in both the overall population [38] and in patients with BMI <20 kg/m^2^ at baseline, when compared with placebo (1.25 kg vs. −0.46 kg; *p* < 0.001). These results highlight anamorelin’s properties as a non-peptide ghrelin analogue to regulate appetite and body weight in patients with advanced cancer [34,35]. Considering that patients with advanced cancer undergo a LBM loss of 0.14–0.20 kg per month [52], the 1 to 2 kg increase observed after the 12-week anamorelin treatment is clinically relevant, and similar to that reported for lung cancer patients recovering from resection surgery *and following high-intensity exercise training* [69]. These results further highlight anamorelin’s efficacy and utility in a patient population with a rapidly deteriorating status due to cancer and associated malnutrition.

Anamorelin was associated with a numerically lower reduction in the co-primary endpoint of handgrip strength, when compared with placebo, although the results did not reach statistical significance (−1.10 kg [95% CI: −1.69, −0.40] vs. −1.58 kg [95% CI: −2.99, −1.14]; *p* = 0.15). This lack of significance may be explained, in part, by the continually worsening condition of these patients with advanced disease, the absence of a training program for these small muscles coupled with the volitional nature of testing that draws on neuromuscular performance, as well as the low mechanical quality of muscle that has been observed in patients with cancer cachexia [70,71]. Moreover, the magnitude of increase in LBM necessary to achieve a detectable shift either in muscle strength or that is discernible to patients is currently unknown. Arguably, maintenance or improvement of function, for example, daily physical activity or level of disability, may provide a better “real-world” measure of the impact of treatments to manage cancer cachexia.

The ACT-ONE trial was a randomized, double-blind, placebo-controlled phase 2 trial that evaluated the efficacy of two different doses of espindolol, an MT-102 anabolic/catabolic transforming agent, in 87 patients with cachexia and stage III/IV NSCLC or colorectal cancer [72]. A statistically significant increase in LBM, the primary efficacy endpoint, was seen after treatment with 10 mg twice-daily (b.i.d.) espindolol compared with placebo (1.76 kg vs. 0.57 kg; *p* = 0.012). Handgrip strength was also significantly improved after administration of 10 mg b.i.d. or 2.5 mg b.i.d. espindolol [72]. The change in handgrip strength following high-dose espindolol was greater than changes observed in stair-climbing power or the 6 min walking test. ACT-ONE was a small phase 2 trial with a heterogeneous patient population, so these results require further confirmation in a phase 3 trial.

The POWER trials were two identically designed randomized, multicenter, multinational phase 3 studies to assess the efficacy of the non-steroidal selective androgen receptor modulator enobosarm for prevention and treatment of muscle wasting in patients with NSCLC initiating first-line chemotherapy [73]. In both trials, enobosarm compared with placebo significantly increased LBM (POWER 1: 0.41 vs. −0.92 kg, *p* = 0.0002; POWER 2: 0.47 vs. −0.37 kg, *p* = 0.0111) at day 84 [74]. However, enobosarm’s effect on physical function was less prominent, as no significant improvements were observed in either of the studies in the percentages of patients gaining ≥10% improvement in stair-climbing power at day 84 (responders; POWER 1: 29.4% vs. 24.2%, *p* = 0.315; POWER 2: 19.5% vs. 24.8%, *p* > 0.05) [74,75]. The co-primary efficacy endpoints of these trials, changes in LBM and physical function (assessed as stair-climbing power) at day 84, were defined after extensive feedback from the United States Food and Drug Administration (FDA). The FDA-informed analysis plan described that LBM, measured at day 84, equal to or higher than the initial LBM of patients is translated into response to treatment [73], citing that “prevention is important, as muscle wasting begins before outward clinical signs or symptoms, including overt weight loss” [76]. However, whether physical function tests (e.g., handgrip strength, sit-to-stand, stair-climbing power) are suitable surrogate measures of strength in patients with advanced cancer and cachexia still needs to be established [77].

In summary, LBM represents a useful tool for assessing the efficacy of pharmacologic interventions for cancer cachexia. Anamorelin has a clinically meaningful effect on body weight and LBM, and the ROMANA studies are among the most relevant in humans to demonstrate a drug’s capacity to reverse loss of lean tissue and LBM in the setting of advanced cancer.

### 2.3. Relevance of Improvement in FM

In patients with cancer cachexia, nutritional therapy to satisfy protein and caloric needs is usually inadequate [54]. By definition, nutritional therapy alone cannot reverse the effects of cancer cachexia [22,78]. One of the underlying consequences, and one of the main characteristics of cancer cachexia, is loss of FM in addition to LBM. FM represents a central location for energy storage, and a diminished accumulation of fatty tissue cannot be reversed by nutritional intervention alone [79], indicating that patients with FM loss have a profoundly altered metabolism.

In the ROMANA trials, anamorelin showed significant benefits in terms of FM, in addition to body weight and LBM (muscle), appendicular LBM, and total body mass. Anamorelin treatment significantly increased FM in both trials, when compared with placebo (ROMANA 1: 1.21 kg [95% CI: −0.2, 2.8] vs. −0.12 kg [95% CI: −0.1, 1.0], *p* < 0.0001; ROMANA 2: 0.77 kg [95% CI: −0.8, 2.4] vs. 0.09 kg [95% CI: −1.1, 1.1], *p* = 0.012) [38]. Notably, the initial observed increase in FM continued beyond week 6 to week 12, suggesting that the response in terms of FM might not have reached a plateau by the end of the study period. These results were confirmed by a pooled *post hoc* analysis of efficacy data from ROMANA 1 and ROMANA 2. In this large data set (*N* = 829), the increase in FM concurred with that reported for the individual trials [80]. Considering that each kilogram of fat contains approximately 9000 kcal, and the average duration of treatment was 12 weeks, which suggests a net positive energy balance of approximately 100 kcal per day. The daily deficit in total energy expenditure of patients with advanced cancer is approximately 100–200 kcal per day [81]. Consequently, the effects of anamorelin on energy balance may be considered clinically meaningful (a net daily difference of more than 200 kcal), as an additional 100 kcal per day would allow most patients to reach normal levels of physical activity or exercise, in line with the American College of Sports Medicine’s recommendations [82].

The observed improvements in FM in anamorelin-treated patients, coupled with improvements in body weight and body composition parameters, suggest a positive effect of anamorelin on food intake. Unfortunately, food intake was not measured in the ROMANA trials. As such, body composition, as measured by changes in the FM and LBM parameters, was used as an endpoint to estimate the influence of anamorelin on energy and protein balance over 12 weeks. This approach was validated by Lieffers et al. [83], who estimated the contributions of organ and tumor mass to whole-body energy demands by monitoring changes in body composition of patients with advanced colorectal cancer and cachexia.

Nevertheless, it remains unclear whether anamorelin improves muscle strength and physical function, or whether such improvement should be required to support the value of increased LBM and FM. Other patient-reported effects might help determine treatment value.

## 3. Patient-Reported Benefits of Anamorelin: The Anorexia/Cachexia Scale

Patients’ emotional well-being and QOL are severely compromised by cancer cachexia and its associated symptoms [39,84,85,86]. Hence, patient-reported measures, which can provide direct evidence of social and clinical outcomes [8] and have independent prognostic value [87], are necessary to assess items of relevance in patients with anorexia/cachexia [88].

The Functional Assessment of Chronic Illness Therapy (FACIT) family of questionnaires is designed to assess QOL. They are among the best-validated instruments for use in patients with cancer [84,89]. The Functional Assessment of Cancer Therapy-General (FACT-G) [90] is the core instrument, and contains four domains: physical, functional, emotional, and social well-being. The FAACT assessment tool combines the FACT-G with the A/CS [39] and has been validated in patients with anorexia/cachexia and advanced cancers [91], including NSCLC [8,39]. The FAACT questionnaire contains the 12-item A/CS domain that specifically addresses anorexia/cachexia (Table 2), and is the ideal tool to measure the complex phenomenon of appetite/eating [92], as the questions not only evaluate cancer anorexia/cachexia symptoms and concerns including appetite, early satiety, and other gastrointestinal symptoms, but also assess patient concerns related to weight/appearance, family interactions, and general health [39]. A treatment specific for cancer anorexia/cachexia can be expected to show an effect on the A/CS domain due to its specificity, but not necessarily on the more general FACT-G. Moreover, a treatment specific for targeting the ghrelin receptor can also be expected to show an effect on the A/CS domain, as this domain contains items related to appetite, eating, and weight loss, which is more proximal to the mechanism of action compared with the general items of the FACT-G.

A recent survey among 95 patients with advanced NSCLC compared QOL and anorexia/cachexia symptom burden in patients with and without considerable weight loss on survey initiation [93]. Considerable weight loss was defined as weight loss >5%, or weight loss >2% in patients already showing depletion according to current body weight and height (BMI <20 kg/m^2^) or skeletal muscle mass (sarcopenia). Patients with considerable weight loss had a significantly lower overall QOL (European Organisation for Research and Treatment of Cancer [EORTC] Quality of Life Questionnaire for palliative cancer care patients [QLQ-C15-PAL] score: 55.2 vs. 66.9; *p* = 0.03); worsened anorexia/cachexia symptoms and concerns (FAACT A/CS domain score: 30.7 vs. 36; *p* = 0.001); and worse fatigue (64.8 vs. 49.1; *p* = 0.007), nausea (19.5 vs. 9.2; *p* = 0.009), and appetite loss (41.0 vs. 23.9; *p* = 0.004). Significantly more patients who lost weight reported moderate or high distress levels than patients whose weight was stable (71% vs. 38%; *p* = 0.007). For patients with considerable weight loss, the most frequently reported symptoms that had the greatest impact on their lives were changes in food taste (38%), fatigue (38%), decrease in appetite (33%), and early satiety (14%) [93]. Notably, food taste, appetite, and early satiety are all items captured in the questions on the A/CS domain, highlighting its utility as an independent tool to measure symptoms and concerns in patients with advanced cancer and cachexia.

When using the FAACT questionnaire and A/CS domain to assess anorexia/cachexia, validated cutoff values (thresholds) for defining patients with anorexia/cachexia and those who respond to treatment are useful. Thresholds chosen to define a responder patient should be determined according to the change in the A/CS domain necessary to establish an important difference (ID) in outcome. Using an anchor-based approach with performance functional status as the anchor [39], a change of four points in the A/CS domain score can be considered an ID. This four-point responder threshold was also confirmed when assessing the psychometric properties of A/CS in NSCLC patients who participated in the ROMANA program using both anchor- and distribution-based approaches [94], and can thus be viewed as a starting point for consideration in any study.

In both ROMANA 1 and ROMANA 2, anamorelin, when compared with placebo, significantly improved anorexia/cachexia symptoms and concerns of patients over the 12-week study period [38,40]. Significant improvements were noted by week 3 and were evident throughout the entire study period. These positive results were confirmed in a post hoc analysis performed in 829 patients from the ROMANA 1 and ROMANA 2 trials [71], where significant improvements in the A/CS domain score were observed over 12 weeks with anamorelin when compared with placebo (treatment difference: 1.84 [95% CI: 0.50, 3.18]; *p* = 0.007). While this comparison of group means between treatment arms aids in determining statistical significance, recent practice for evaluating clinical meaningfulness focuses on interpretation of treatment benefit at the individual level (i.e., responder analysis). As such, in the pooled overall population, a significantly higher proportion of patients achieved a clinically meaningful improvement in the A/CS domain score following anamorelin than following placebo (50% vs. 37%; *p* < 0.001) [71]. In patients with BMI <20 kg/m^2^ at baseline treated with anamorelin, the A/CS improvement rate (59%) was higher than in the overall patient population, whereas the placebo arm had a rate similar to the overall population (34%). These results further highlight anamorelin’s efficacy in patients with severe weight loss. 

Anamorelin also led to improvements in several individual items of the A/CS in the overall population (Table 3). A retrospective analysis showed that, in patients with BMI <20 kg/m^2^ at baseline, the effect of anamorelin versus placebo on several single items of the A/CS domain was more pronounced than in the overall population. The two items most changed in the overall population (“*I am concerned about how thin I look*” and “*I am worried about my weight*”; Table 2) reflect important concerns expressed by patients due to the physical and psychosocial effects of cancer-associated anorexia and weight loss, which are ameliorated by anamorelin treatment. Importantly, the reported improvements in symptom burden were consistently maintained throughout the 12-week safety extension study ROMANA 3 [41,95]. Over the entire 24-week treatment period, anamorelin led to improvements in anorexia/cachexia symptoms, with significant treatment differences at weeks 3, 6, 9, 12, and 16 (*p* < 0.05). A post hoc analysis confirmed these results and reported that, over the entire 24-week treatment period, patients receiving anamorelin had a larger mean increase in their A/CS domain score compared with placebo (4.5 [95% CI: 2.7, 6.3] vs. 3.2 [95% CI: 1.0, 5.2]).

In summary, these results on the relevant patient-reported outcomes highlight anamorelin’s benefit over an extended period, overall and in a patient population with compounding health issues due to progressive disease.

## 4. Overall Considerations

The reported improvements in LBM, combined with enhancements in total body weight, FM, and anorexia/cachexia symptoms and concerns, suggest that anamorelin not only stimulates food intake, but also supports its conversion into energy storage. This is among the first proof in humans of a drug’s capacity to reverse loss of muscle tissue and LBM. Furthermore, the evident arrest of ongoing weight loss and switch from negative to positive energy balance reported in patients from the ROMANA trials constitutes further evidence of anamorelin’s efficacy in patients with advanced cancer and cachexia. Importantly, the fact that anamorelin treatment results in increased body weight/LBM, while also improving anorexia/cachexia-related symptoms and associated patient perceptions, is in alignment with published literature from international medical community consensus that supports body weight maintenance/gain and symptom improvement as important and meaningful treatment goals for cancer anorexia/cachexia [26,27]. The results also bear crucial relevance, as these patients represent an at-risk population with increased difficulty to treat as a result of their compounding advanced disease and associated comorbidities.

## 5. Expert Statement

**Figure 2:**
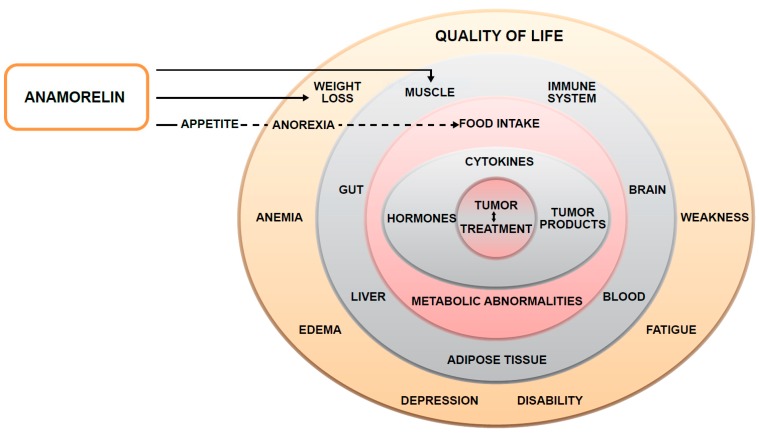
Schematic of the complex, multilayered, and multi-organ nature of cancer cachexia and the layers targeted by anamorelin in this process.

Cancer cachexia is a highly complex, multilayered syndrome, with its core consisting of the interaction between tumor cells and the pharmacologic treatments given to control the tumor (Figure 2). Key cellular and soluble mediators, activated as a result of this interaction, together with the neuroendocrine system, induce alterations in systemic metabolism and food intake. These alterations then result in changes in the mass and functionality of various organs and tissues, which ultimately translate into diminished QOL (Figure 2), increased morbidity, and reduced survival.

While the most efficient cancer cachexia treatment would be curing the cancer itself, which would address all the layers detailed above, for most solid malignancies, this remains an unrealistic goal. Furthermore, targeting the activated key mediators has also proven largely ineffective as a result of their heterogeneity and redundancy, which renders single-mediator treatments clinically unsuccessful. As such, the most beneficial approach is to target mechanisms [2,29], an approach where anamorelin has the advantage of stimulating both appetite and possibly food intake, as well as the potential to promote anabolism and, therefore, a significant gain of muscle mass (Figure 2). In this context, the effects of anamorelin treatment on the key diagnostic features of cancer cachexia, namely, body weight, body composition parameters, and symptom burden, bear crucial relevance. In the ROMANA studies, mean weight increased by 1.8 kg in patients randomized to anamorelin over 12 weeks, as compared with a mean weight loss of −0.4 kg in patients randomized to placebo. Body composition analysis suggested that anamorelin is an active anabolic agent in patients with NSCLC. It is noteworthy that anamorelin also induced a meaningful benefit on the main patient-reported outcome of anorexia/cachexia symptoms/concerns.

In summary, the ROMANA trials have provided valuable knowledge, thus highlighting the therapeutic effects of anamorelin as an initial, but highly significant, step [96] toward achieving overall management of cancer cachexia through future multimodal treatment. 

## Figures and Tables

**Table 1:** Major results of the ROMANA 1 and ROMANA 2 studies.

Parameter	Anamorelin vs. Placebo	
	ROMANA 1	ROMANA 2
Median LBM. kg	0.99 vs. −0.47	0.65 vs. −0.98
Median HGS, kg	−1.10 vs. −1.58	–1.49 vs. −0.95
Mean body weight, kg	2.20 vs. 0.14	0.95 vs. −0.57
Median FM, kg	1.21 vs. −0.12	0.77 vs. 0.09
Median aLBM, kg	0.87 vs. 0.30	0.62 vs. −0.21
Mean FAACT A/CS domain score	4.12 vs. 1.92	3.48 vs. 1.34

aLBM, appendicular lean body mass; FAACT A/CS, Functional Assessment of Anorexia/Cachexia Therapy Anorexia/Cachexia Subscale; FM, fat mass; HGS, handgrip strength; LBM, lean body mass; TBM, total body mass.

**Table 2:** The 12 items of the FAACT A/CS domain.

I have a good appetite
The amount I eat is sufficient to meet my needs
I am worried about my weight
Most food tastes unpleasant to me
I am concerned about how thin I look
My interest in food drops as soon as I try to eat
I have difficulty eating rich or “heavy” foods
My family or friends are pressuring me to eat
I have been vomiting
When I eat, I seem to get full quickly
I have pain in my stomach area
My general health is improving

A/CS, Anorexia/Cachexia Subscale; FAACT, Functional Assessment of Anorexia/Cachexia Therapy. Each statement is rated for a period of the past seven days, and is answered on a five-point rating scale ranging from “not at all” to “very much” (www.facit.org).

**Table 3:** Anamorelin treatment effect size on the 12 individual items of the FAACT A/CS domain.

Individual Item	Overall Patient Population			Patients with BMI <20 kg/m^2^ at Baseline		
	Mean (95% CI)	Standardized Effect Size	*p* Value	Mean (95% CI)	Standardized Effect Size	*p* Value
I am concerned about how thin I look	0.41 (0.218, 0.601)	0.317	0.000	0.53 (0.083, 0.976)	0.375	0.018
I am worried about my weight	0.29 (0.086, 0.493)	0.213	0.005	0.37 (–0.091, 0.831)	0.253	0.110
My family or friends are pressuring me to eat	0.23 (0.025, 0.454)	0.163	0.029	0.72 (0.253, 1.186)	0.424	0.002
The amount I eat is sufficient to meet my needs	0.16 (–0.011, 0.331)	0.139	0.064	0.54 (0.168, 0.911)	0.451	0.005
Most food tastes unpleasant to me	0.16 (–0.036, 0.356)	0.117	0.117	0.39 (–0.072, 0.852)	0.254	0.109
I have pain in my stomach area	0.15 (–0.001, 0.301)	0.149	0.046	0.36 (–0.057, 0.777)	0.273	0.085
I have a good appetite	0.12 (–0.063, 0.303)	0.094	0.210	0.31 (–0.115, 0.735)	0.218	0.167
My interest in food drops as soon as I try to eat	0.11 (–0.075, 0.295)	0.085	0.256	0.6 (0.165, 1.034)	0.424	0.008
I have been vomiting	0.08 (–0.054, 0.214)	0.088	0.239	0.2 (–0.1, 0.5)	0.206	0.193
When I eat, I seem to get full quickly	0.04 (–0.148, 0.228)	0.036	0.630	0.46 (–0.037, 0.957)	0.302	0.057
My general health is improving	0.04 (–0.148, 0.228)	0.032	0.669	0.35 (–0.095, 0.795)	0.256	0.106
I have difficulty eating rich or “heavy” foods	0.02 (–0.187, 0.227)	0.015	0.845	0.44 (–0.038, 0.918)	0.297	0.061

A/CS, Anorexia/Cachexia Subscale; BMI, body mass index; CI, confidence interval; FAACT, Functional Assessment of Anorexia/Cachexia Therapy.

## Abbreviations

A/CS	Anorexia/Cachexia Subscale
ADL	Activities of daily living
AEs	Adverse events
b.i.d.	Twice daily
BMI	Body mass index
CI	Confidence interval
EORTC QLQ-C15-PAL	European Organisation for Research and Treatment of Cancer Quality of Life Questionnaire for palliative cancer care patients
FAACT	Functional Assessment of Anorexia/Cachexia Therapy
FACIT	Functional Assessment of Chronic Illness Therapy
FACT-G	Functional Assessment of Cancer Therapy-General
FDA	United States Food and Drug Administration
FM	Fat mass
GH	Growth hormone
ID	Important difference
LBM	Lean body mass
NSCLC	Non-small cell lung cancer
QOL	Quality of life
